# Clinical Utility of Multicancer Detection in Symptomatic Patients: A Decision-Making Perspective

**DOI:** 10.1200/PO-25-00279

**Published:** 2025-12-05

**Authors:** Giuliano Netto Flores Cruz, Keegan Korthauer

**Affiliations:** ^1^ Centre for Molecular Medicine and Therapeutics, British Columbia Children's Hospital Research Institute, Vancouver, Canada; ^2^ Department of Statistics, The University of British Columbia, Vancouver, Canada; ^3^ BiomeHub, Florianópolis, Brazil

## Abstract

**PURPOSE:**

There is growing interest in multicancer detection (MCD) blood tests for diagnosing patients with cancer-related symptoms. However, recent studies suggest that MCD testing may not be sensitive enough to rule out cancer in the symptomatic population without retraining the underlying classifiers. On the basis of clinical guidelines for suspected cancer referral, here we cast these data into a formal diagnostic decision-making perspective to assess clinical utility.

**METHODS:**

Data were extracted from the SYMPLIFY study (ISRCTN10226380), which evaluated the performance of the Galleri test (GRAIL, LLC). The decision threshold for suspected cancer referral was extracted from the National Institute for Health and Care Excellence Guideline 12. Clinical utility was estimated using Bayesian decision curve analysis.

**RESULTS:**

For the guideline-derived decision threshold of 3%, the Galleri MCD test avoided 18,005 unnecessary suspected cancer referrals per 100,000 symptomatic patients, with a 99.4% posterior probability of clinical utility. High probabilities of clinical utility were observed for gynecologic, lower GI, and upper GI referral pathways, avoiding between 25,414 and 62,501 unnecessary referrals per 100,000 symptomatic patients. The rapid diagnostic center and lung referral pathways showed negligible probabilities of clinical utility. The minimum diagnostic performance required for clinical utility varied significantly across referral pathways. The gynecologic pathway showed the lowest sensitivity requirement (under 30% for a highly specific test) and the lung pathway the highest (over 90% for any specificity level).

**CONCLUSION:**

Clinical utility of MCD testing for symptomatic patients in the United Kingdom varies substantially across referral pathways but is favorable for gynecologic and GI cancers. Future pathway-specific optimization of MCD tests must consider clinical utility explicitly and does not require retraining the underlying machine learning classifiers.

## INTRODUCTION

Multicancer detection (MCD) testing has the potential to revolutionize cancer diagnosis.^1,2^ The Galleri test (GRAIL, LLC) is a blood-based MCD test primarily developed for cancer screening in asymptomatic individuals.^3^ Using a targeted assay, plasma cell-free DNA methylation measurements are obtained and fed into a machine-learning algorithm trained to detect the presence of a cancer signal. If a cancer signal is detected, the tissue of origin is also predicted. Previous studies indicated that Galleri detects cancer with high specificity and moderate sensitivity, while classification of cancer signal origin was considered reasonably accurate.^4^

**CONTEXT**

**Key Objective**
Is the Galleri multicancer detection (MCD) test clinically useful for ruling out disease in patients presenting to primary care with cancer-related symptoms?
**Knowledge Generated**
Considering decision thresholds encoded in the National Institute for Health and Care Excellence Guideline 12 (NG12), the Galleri MCD test showed high probabilities of clinical utility for patients under suspicion of gynecologic and GI cancers (upper and lower) in a large prospective cohort in the United Kingdom. Minimum sensitivity and specificity required for clinical utility varied significantly across cancer referral pathways established in the NG12.
**Relevance**
The Galleri MCD test can be clinically useful in its current form for ruling out gynecologic and GI cancers in symptomatic patients presenting to primary care in the United Kingdom.

Recently, MCD in symptomatic populations has also gained attention.^5^ Beyond screening, the hope is that MCD may help guide diagnostic decision making for patients presenting to primary care with cancer-related symptoms. One major advantage is the potential reduction in unnecessary suspected cancer referrals, avoiding exposure of cancer-free patients to the negative consequences of downstream diagnostic workups that are often invasive, costly, and psychologically distressful. In a retrospective analysis of the Circulating Cell-free Genome Atlas (CCGA) study, Bryce et al^6^ reported promising results for the Galleri test in a subgroup of participants with symptoms suspicious of cancer. Highlighted in an accompanying editorial by Wang and Tie,^5^ this was the first study to suggest that MCD testing could be clinically useful in the symptomatic population. The editorial also highlighted early results from the SYMPLIFY study (ISCRTN10226380), a large investigation specifically designed to assess this application of the Galleri test, reported at the 2023 ASCO Annual Meeting.

Now published, SYMPLIFY was a prospective cohort study in the United Kingdom that enrolled over 6,000 symptomatic individuals referred from primary care for cancer investigation.^7^ On the basis of the National Institute for Health and Care Excellence (NICE) Guideline 12 (NG12)—suspected cancer: recognition and referral,^8^ five referral pathways were included, depending on the type of suspected cancer: gynecologic, lung, upper GI, lower GI, and nonspecific (rapid diagnostic center [RDC]). Overall, 6.7% of participants in the SYMPLIFY study had a confirmed cancer diagnosis after a 9-month follow-up.^7^ The lung cancer pathway had the highest cancer proportion (29.8%) and the gynecologic pathway had the lowest (3.7%). The specificity of the Galleri test was consistently above 96% for all pathways. However, sensitivity varied from 49.1% (gynecological) to 80.4% (upper GI), with an overall estimate of 66.3%. These results led both the SYMPLIFY authors and Wang and Tie^5^ to suggest that optimization of the test for symptomatic populations is required for implementation.^7^ Although justifiable, such an interpretation does not formally assess the clinical utility of the observed diagnostic performance.

In this work, we adopt a decision-making perspective to evaluate the clinical utility of the Galleri test in preventing unnecessary cancer investigations for symptomatic populations in the United Kingdom. We then discuss strategies for MCD test optimization that consider clinical utility explicitly. Our approach reveals that (1) the Galleri test may already be clinically useful in its current form for both GI and gynecologic cancers; and (2) the test can be further improved with pathway-specific, clinical utility–aware optimization without retraining the underlying classifiers. We finish by discussing the implications for implementation and further research.

## METHODS

### Study Population

We included data from 5,461 adult participants of the SYMPLIFY study who were referred for urgent investigation of a suspected cancer and reached a diagnostic resolution within a 9-month follow-up. Approximately 75% of patients were between age 50 and 79 years, with 66.1% female and 90.4% White. Referral criteria were based on NG12 (for symptoms suggestive of gynecologic, lung, and lower or upper GI cancers) and the UK's National Health Service (NHS) RDC specification (for nonspecific symptoms). For example, NG12 recommends that primary care physicians refer women age 55 years and older with postmenopausal bleeding through the gynecologic pathway for suspected endometrial cancer. As another example, the RDC pathway involves specialized clinics to investigate cancer at multiple sites because of nonspecific persistent symptoms, including unexplained weight loss, fatigue, and nausea.^7^

### Diagnostic Performance Data Extraction

Diagnostic performance data were extracted from the SYMPLIFY study publication (abstract and supplementary table on p. 10 of the supplementary data).^7^ We extracted raw counts for sample size (N), the number of participants with cancer (D), and the number of positive MCD tests (P), as well as point estimates of sensitivity (Sens), specificity (Spec), positive predictive value, and negative predictive value (NPV). We used the extracted statistics to compute the corresponding raw counts of true positives (TPs), true negatives (TNs), false positives (FPs), and false negatives (FNs) since these raw counts were not explicitly reported in the SYMPLIFY article. TP and TN counts were computed, respectively, as TP=Sens×D and TN=Spec×(N−D). FP and FN counts were computed, respectively, as FP=N−D−TN and FN=D−TP. To mitigate rounding errors in the overall pathway only, the TN counts were computed as TN=floor(NPV×(N−P)) where the floor function rounds its input to the lowest integer. Point estimates and confidence intervals on the basis of the computed counts matched exactly their corresponding values reported in the SYMPLIFY study.

### The Net Benefit of MCD Testing

Beyond diagnostic performance, the clinical utility of MCD testing depends highly on the clinical decision for which the test is intended.^9^ For symptomatic patients presenting to primary care, NG12 establishes criteria for suspected cancer investigation that balance the tradeoff between the risk of missing true cancers and the cost of referral overload and potential overdiagnosis.^8^ In light of both clinical and health economic context, this tradeoff was operationalized in NG12 as a set of diagnostic criteria that identify patients whose risk of cancer is at least 3%.^10^ From a decision-making perspective, a risk threshold of 3% approximates the relative value of potential diagnostic outcomes as assessed by the guideline development group.^11^ In this setting, a patient perceived to have at least a 3% probability of cancer is referred because the harm of an unnecessary referral weighs relatively little compared with the benefit of a timely diagnosis.^10,12^ Thus, further stratifying referral-eligible patients on the basis of MCD testing may be helpful to the extent that it reduces unnecessary referrals while still respecting the tradeoff encoded in the clinical guidelines.

In this context, the net benefit of MCD testing to avoid unnecessary referrals can be represented by the rate of net TNs provided by the test among referral-eligible patients.^11^ Although a TN MCD test avoids an unnecessary referral, a FN test delays the diagnosis of a true cancer. Given a risk threshold t, the number of net TNs is a weighted subtraction between TNs and FNs (Eq 1):

(1) $$\text{Net}\;\text{TNs}=\text{TN}-\left(1-t\right)/t\times \text{FN}$$

where TN and FN are the numbers of true negatives and false negatives, respectively, obtained from a diagnostic accuracy study such as the SYMPLIFY. A risk threshold of *t* = 3% thus mathematically yields a relative cost of (100 − 3)/3 = 32.3 for each FN, implying that failing to detect a cancer is 32.3 times worse than performing unnecessary diagnostic procedures.^13^ Net TPs are similarly defined (Eq 2):

(2) $$\text{Net}\;\text{TPs}=\text{TP}-t/\left(1-t\right)\times \text{FP}$$

where TP and FP are the numbers of true positives and false positives, respectively. Should the Galleri test be implemented in the symptomatic setting, the net benefit for referred patients is quantified as a rate of net TPs, whereas the net benefit for the unreferred is quantified in terms of net TNs.^14,15^ The two formulas above come from formal decision analysis and lead to equivalent conclusions: the decision strategy that maximizes net TPs also maximizes net TNs.^13^ However, in this case, quantifying net TNs allows the natural interpretation of unnecessary referrals avoided.

In a study with n individuals, decision curve analysis (DCA) results from plotting the net benefit, defined as net TPs (or TNs) divided by n, for a range of clinically relevant risk thresholds representing varying referral preferences. For instance, a preference for more aggressive referral than current guidelines may be encoded as a risk threshold lower than 3%. However, because modifying the threshold modifies referral criteria and, thus, the target population for MCD testing, here we focus on the risk threshold of 3% currently encoded in NG12.

### Bayesian DCA

Bayesian DCA was performed using the BayesDCA R package (v.0.0.0.9).^16^ For each decision threshold, BayesDCA estimates the joint posterior distribution of sensitivity (Se), specificity (Sp), and prevalence (p) using a Bayesian binomial model with Beta prior for each parameter. Using 10,000 draws from the joint posterior distribution, the number of net TNs and TPs per 100,000 patients was estimated as Sp×(1−p)−(1−t)/t×p×(1−Se)×100,000 and Se×p−t/(1−t)×(1−p)×(1−Sp)×100,000, respectively. We used default Beta(1, 1) priors (uniform between 0 and 1) for all parameters. All analyses were performed in R (v. 4.3.0).

## RESULTS

### Galleri's Utility to Avoid Unnecessary Cancer Referrals

**FIG 1.**
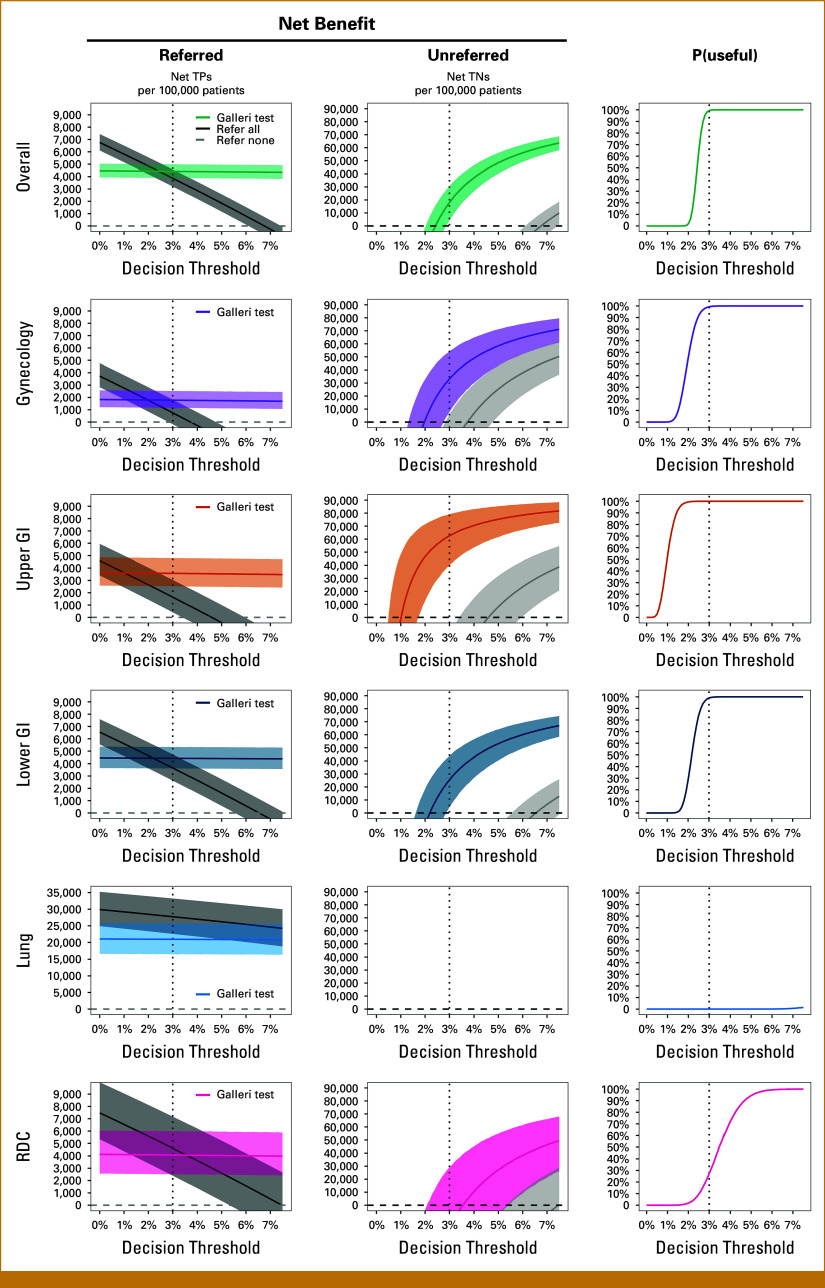
The clinical utility of the Galleri test to avoid unnecessary suspected cancer referrals of symptomatic patients strongly depends on the referral pathway. Net benefit was computed in terms of net TPs (referred, left) or net TNs (unreferred, middle) using Bayesian DCA applied to the data from the SYMPLIFY study. For each decision threshold, the probability that the Galleri test is clinically useful was defined as the posterior probability that the test provides a net benefit greater than referring all or referring no patients (P[useful], right). The net benefit for the unreferred can be interpreted as unnecessary referrals avoided per 100,000 symptomatic patients, already accounting for the harm of any FNs. Results are shown for all SYMPLIFY participants (overall, pooled across all pathways) and stratified by referral pathway. Based on NG12, the decision threshold of 3% is highlighted as a dashed vertical line. Solid lines and shaded areas are posterior means and 2.5%/97.5% posterior percentiles, respectively (left and middle columns). DCA, decision curve analysis; FN, false negative; NG12, National Institute for Health and Care Excellence Guideline 12; RDC, rapid diagnostic center; TN, true negative; TP, true positive.

The risk threshold encoded in NG12 allows for interpreting Galleri's diagnostic performance from a decision-making perspective. Enabled by our Bayesian approach to DCA, Figure 1 shows the net benefit of referred and unreferred patients alongside the estimated probability that the Galleri MCD test is clinically useful, defined as the probability that Galleri has the highest net benefit among all decision strategies considered.

Pooling across all referral pathways, the Bayesian DCA estimate of the posterior probability that the Galleri MCD test is clinically useful for deciding whether to refer symptomatic patients for cancer investigation was >99.4% for the 3% risk threshold. At this risk threshold, we estimated that the MCD test avoids 18,005 (95% credible interval [CrI], 4,231-30,677) unnecessary referrals per 100,000 symptomatic patients. Probabilities of clinical utility and net numbers of unnecessary referrals avoided per 100,000 patients were higher at higher risk thresholds. Although there was considerable uncertainty for thresholds between 2% and 3%, referring all eligible patients was the best strategy with risk thresholds at or below 2%.

However, pathway-specific clinical utility was substantially heterogeneous. At the risk threshold of 3%, a high posterior probability of clinical utility was observed for the gynecologic, upper GI, and lower GI referral pathways, with net numbers of unnecessary referrals avoided per 100,000 of 33,121 (CrI, 7,704-54,131), 62,501 (CrI, 40,195-79,175), and 25,414 (CrI, 4,409-43,611), respectively. Although greater utility was observed under higher risk thresholds for most pathways, the upper GI pathway was the only one in which the Galleri test showed a high probability of clinical utility for the 2% threshold (99.4%, 46,628 [CrI, 12,738-71,481] unnecessary referrals avoided per 100,000 patients). There was also considerable uncertainty for the upper GI pathway for thresholds between 1% and 2%, and negligible probabilities of clinical utility for all pathways at thresholds lower than 1%.

For the lung cancer referral pathway, we observed virtually zero posterior probability of clinical utility from using the Galleri MCD test to guide referral decisions across all risk thresholds examined: referring all eligible patients was the best decision strategy available. More uncertainty was observed for the nonspecific referral pathway (RDC), where probabilities of clinical utility ranged from nearly zero at the 2% threshold to 94.4% at the 5% threshold. For the risk threshold of 3%, we estimated a probability of clinical utility of only 27.4%.

### Sensitivity and Specificity Define Clinical Utility Regions

The Galleri test showed practically no chance of being clinically useful for the lung pathway in the SYMPLIFY study population. This can be explained by the implied relationship between test performance and clinical utility in a context of high prevalence and low risk threshold. Notice that the net benefit for the unreferred (net TNs divided by n) can be rewritten as a function of specificity (Sp), sensitivity (Se), and prevalence (p) as follows (Eq 3):

$${\text{Net}\;\text{benefit}}_{\text{unref}.}=\text{Sp}\times \left(1-p\right)-\left(1-t\right)/t\times p\times \left(1-\text{Se}\right)$$ (3)

**FIG 2.**
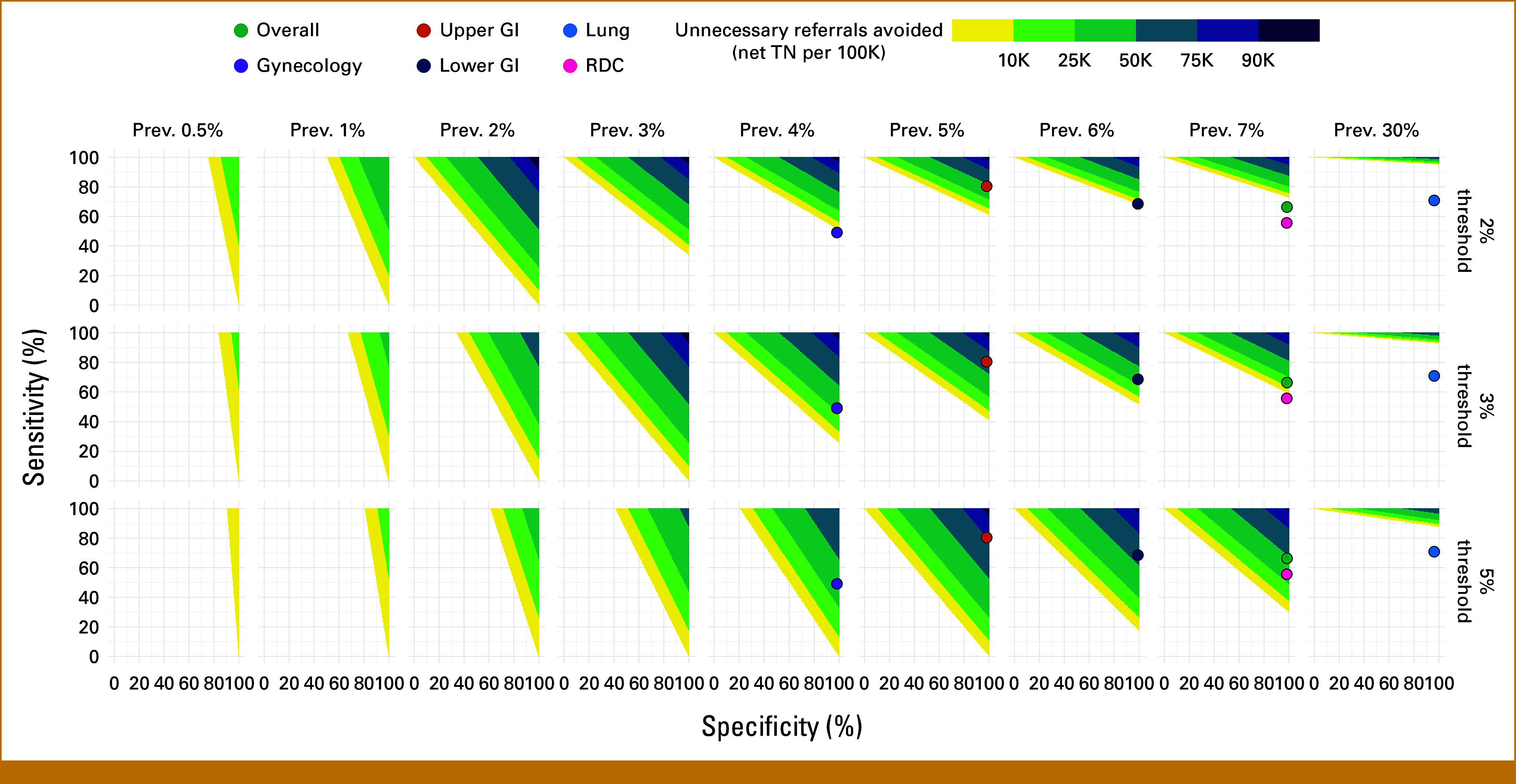
Risk threshold and outcome prevalence define regions of clinical utility for diagnostic test performance. Given a risk threshold and an outcome prevalence, colored areas show corresponding regions of clinical utility, defined as sets of sensitivity and specificity values a diagnostic test must have to be clinically useful. Here, clinically useful means having greater net benefit than the default strategies of referring all or no patients. Higher sensitivity and specificity lead to greater utility, quantified in terms of net TNs per 100,000 symptomatic patients. Diagnostic tests outside clinical utility regions are worse than the corresponding default strategy and, hence, are not clinically useful. The dots represent the observed performance for the Galleri test in the SYMPLIFY study, with approximate prevalences. RDC, rapid diagnostic center; TN, true negative.

Hence, a given cancer prevalence and a choice of risk threshold define regions of sensitivity and specificity that yield net benefit gain. Figure 2 illustrates such clinical utility regions. Settings with higher prevalence require MCD tests with higher sensitivity to achieve clinical utility, in particular when the prevalence is higher than the risk threshold. Low-prevalence settings require more specificity, especially when the prevalence is lower than the threshold (eg, screening of asymptomatic individuals).

We can now understand how good an MCD test needs to be to outperform the current strategy of referring all symptomatic patients from the lung pathway. Notice that referring all patients yields 0% specificity and 100% sensitivity, leading to zero net benefit for the unreferred. Suppose the MCD test is 100% specific. Then, a prevalence of 30% and a risk threshold of 3% imply that the minimum sensitivity required to achieve clinical utility is given by solving 100%×(1%−30%)−(1%−3%)/3%×30%×(1−Se)>0 for Se. Thus, an unrealistically specific MCD test that generates zero FPs still needs a sensitivity > 92.8% to be clinically useful. Lower specificity will lead to even higher sensitivity requirements. Therefore, for the lung pathway, any optimized version of the Galleri MCD test must achieve a very high sensitivity to be clinically useful. Conversely, for the gynecologic pathway, a highly specific MCD test with sensitivity as low as 30% can already be clinically useful.

### Optimizing the Galleri Test for Clinical Utility in the Symptomatic Population Without Retraining

**FIG 3.**
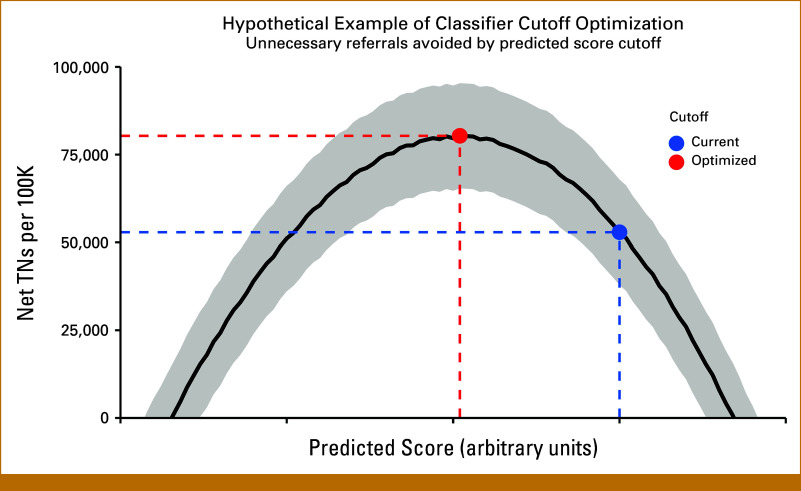
Maximizing the clinical utility of MCD tests by optimizing classifier cutoff. On the basis of logistic regression, the Galleri MCD test is a classifier that could use different cutoffs in the underlying continuous score for assigning a positive cancer signal. Using external validation data, the clinical utility of a hypothetical MCD test can be maximized by choosing the cutoff value in the underlying predicted score that maximizes net TNs per 100,000 patients. Each cutoff value yields a potential classifier, which is assessed as a binary test using DCA with the decision threshold fixed at 3%, following the NICE guidelines. This maximizes the clinical utility of MCD testing for the symptomatic population while circumventing the need to fix arbitrary sensitivity or specificity cutoffs. Ideally, the updated classifier is then validated on new data. This approach is inspired by the proposal of Mishra et al.^17^ The solid line and shaded area are hypothetical posterior mean and 2.5%/97.5% posterior percentiles, respectively. The performance of the hypothetical MCD test before cutoff optimization is shown as a blue point, while the red point represents the performance of the optimized classifier. DCA, decision curve analysis; MCD, multicancer detection; NICE, National Institute for Health and Care Excellence; TN, true negative.

Both the SYMPLIFY authors and Wang and Tie^5^ suggested that optimization of the Galleri test for symptomatic populations is required.^5,7^ Yet, beyond increasing NPV, how to perform such optimization is unclear. Because of heterogeneity in both test performance and cancer prevalence, pathway-specific MCD test optimization is required to maximize clinical utility. Using the SYMPLIFY data, this could be achieved without retraining the underlying classifiers by picking the cutoff value for the underlying predicted score that directly maximizes net TNs,^17^ as shown in Figure 3.

## DISCUSSION

MCD testing offers a promising screening strategy targeted at asymptomatic populations. To our knowledge, the SYMPLIFY study was the first large-scale study prospectively assessing the performance of a MCD test in symptomatic individuals.^7^ The estimated high specificity and modest sensitivity led the authors to conclude that the test requires optimization before it can be clinically useful in avoiding unnecessary suspected cancer referrals. The one exception was the upper GI referral pathway, in which the highest sensitivity was observed (80.4%). In this work, we added nuance to these conclusions by formally assessing clinical utility.

Although there is always some degree of subjectivity regarding what test performance is enough to justify implementation, the NG12 defines the risk threshold of 3% as a reasonable consensus between patient and clinician preferences and health economic consequence.^8,12,18-23^ The risk threshold captures the clinical context by representing the relative preferences regarding the benefit of a TN and the harm of a FN.^11^ Using Bayesian DCA and the guideline-derived risk threshold of 3%, we have shown that the SYMPLIFY data support the clinical utility of the Galleri MCD test in symptomatic patients from the gynecologic, upper GI, and lower GI referral pathways. This is in contrast to the overall interpretation from the SYMPLIFY authors, who interpreted Galleri's observed sensitivity as generally insufficient to rule out referral, with the upper GI pathway being the only exception.^7^

The above result holds although the Galleri MCD test showed the lowest sensitivity in the gynecologic pathway (49.1%), with a negative likelihood ratio (NLR) of 0.52.^7^ This can be explained by the fact that clinical utility depends on the clinical context (through prevalence and decision threshold) and the test performance (through sensitivity and specificity). As a result, a negative test in the gynecologic pathway brings a pretest probability (ie, prevalence) of 3.7% down to a post-test probability of 1.9%,^7^ which is below the decision threshold of 3% and therefore potentially enough to rule out referral. By contrast, a sensitivity of 70.8% and an NLR of 0.3 were not enough to render the test useful in the lung pathway, in which a negative result reduced a pretest probability of almost 30% down to 11.4%, well above the decision threshold.

The risk threshold of 2% is an important alternative to the NICE threshold as it provides the possibility of detecting more cancers with relatively modest resource implications.^24^ Such liberalization of referral criteria is consistent with reported patient preferences.^19^ It may also be relevant to patients with comorbidities due to a worse prognosis if they have cancer, or patients with more years of life to be gained from successfully detecting a cancer early (eg, children).^8,25^ At the 2% threshold, only the upper GI had a high probability of clinical utility. At risk thresholds between 3% and 7%, the Galleri MCD test showed a high probability of clinical utility for the gynecologic, upper GI, and lower GI pathways. These higher risk thresholds represent more restrictive referral criteria, more closely aligned with past versions of the NG12.^8,20^ However, it is important to note that modifying the risk threshold for suspected cancer referrals also modifies the population of patients who are eligible for referral, potentially leading to worse MCD test performance. In such a case, the estimates of sensitivity and specificity from the SYMPLIFY study may no longer be applicable.^26^

The utility of the Galleri test in avoiding unnecessary referrals needs to be considered in light of its financial costs. Considering the uncertainty intervals observed in the gynecologic and GI pathways, the net number of unnecessary referrals avoided by using the MCD test may range from about 4,000 to 80,000 referrals per 100,000 patients. These numbers may have different implications for different health care systems, given the Galleri test's 2-week turnaround time and current listed price of roughly $950 US dollars per test.^7,27^

The SYMPLIFY authors suggested optimizing the Galleri's test for NPV.^7^ However, it is unclear how much negative (or positive) predictive value is required without an objective definition of clinical utility. Moreover, in the context of precision oncology, there is little justification for ignoring variable clinical utility of MCD tests across cancer referral pathways. Here, we suggest choosing pathway-specific cutoffs for a positive MCD test by directly maximizing the net number of unnecessary referrals avoided per 100,000 patients, without necessarily retraining the underlying classifiers.

Finally, as we leveraged SYMPLIFY's data to assess clinical utility, our conclusions primarily hold for symptomatic individuals presenting to primary care in the United Kingdom. More evidence from this population is unlikely to change our main conclusions, as the probabilities of clinical utility at the 2%-3% thresholds were mostly near 0% or 100%. Yet, to our knowledge, since SYMPLIFY is currently the only prospective cohort study in this setting, more studies are needed in similar populations from different geographic locations and health systems. For example, Bryce et al^6^ reported estimates of Galleri's sensitivity (64.3%) and specificity (99.5%) for symptomatic patients in the United States. These were similar to SYMPLIFY's overall estimates (66.3% and 98.4%, respectively), which suggests that MCD test performance for symptomatic patients may be relatively consistent across the United States and the United Kingdom. However, CCGA's case-control design precludes estimation of cancer prevalence, which also requires accounting for differences in referral practices between the two countries. As a result, any sensitivity and specificity estimates need to be considered together with context-dependent pretest probabilities. In this setting, clinical utility regions may provide a framework for reasoning about potential utility.

In conclusion, we showed that the Galleri MCD test may be clinically useful in its current form for the gynecologic, upper GI, and lower GI referral pathways of the NHS. In the lung pathway, a substantially higher sensitivity is required for clinical utility. Pathway-specific optimization of MCD tests, considering clinical utility explicitly instead of performance metrics only, may result in greater net rates of unnecessary referrals avoided, potentially making it useful under further liberalization of the referral criteria.

## Data Availability

Data and code required to reproduce this manuscript are publicly available at https://github.com/giulianonetto/mcd-clinical-utility.
